# Neuroprotective Effects of a Novel Tetrapeptide SGGY from Walnut against H_2_O_2_-Stimulated Oxidative Stress in SH-SY5Y Cells: Possible Involved JNK, p38 and Nrf2 Signaling Pathways

**DOI:** 10.3390/foods12071490

**Published:** 2023-04-01

**Authors:** Li Feng, Yingmin Wu, Jiankang Wang, Yuting Han, Junrong Huang, Huaide Xu

**Affiliations:** 1 School of Food Science and Engineering, Natural Food Macromolecule Research Center, Shaanxi University of Science & Technology, Xi’an 710021, China; fengli0304@sust.edu.cn (L.F.);; 2 College of Food Science and Engineering, Northwest A&F University, Yangling 712100, China

**Keywords:** SGGY, oxidative stress, antioxidant, SH-SY5Y cells, signaling pathways

## Abstract

SGGY, an antioxidant tetrapeptide identified from walnut protein hydrolysate in our previous study, has been suggested to possess the potential to alleviate oxidative stress in cells. In this paper, the neuroprotective effects of SGGY on H_2_O_2_-stimulated oxidative stress in SH-SY5Y cells and the underlying mechanisms were investigated. Results showed that SGGY alleviated H_2_O_2_-induced oxidative stress by decreasing the intracellular reactive oxygen species (ROS) level and altering the mitochondrial membrane potential (MMP), thereby inhibiting apoptosis and increasing cell viability. SGGY significantly restored antioxidant enzyme activities and reduced malondialdehyde (MDA) content accordingly. Moreover, SGGY promoted the nuclear translocation of nuclear factor erythroid 2-related factor 2 (Nrf2) and suppressed the H_2_O_2_-induced activation of JNK and p38 mitogen-activated protein kinases (MAPKs). Taken together, these results suggested that SGGY protected SH-SY5Y cells from H_2_O_2_-provoked oxidative stress by enhancing the ability of cellular antioxidant defense, and the possible mechanism involved MAPKs and Nrf2 signaling pathways.

## 1. Introduction

Neurodegenerative diseases always characterized by cognitive decline are identified as a major cause of death in the elderly population worldwide [1,2]. Oxidative stress, the imbalance between excessive reactive oxygen species (ROS) and limited scavenging capacity, has been established as a contributing element to the pathogenesis of neurodegenerative diseases [3]. A possible reason is that the brain is susceptible to oxidative damage, which often occurs in the early stages of neurodegenerative diseases [4]. Moreover, oxidative stress can lead to brain injury by increasing the products of lipid peroxidation and the oxidation of proteins and DNA, which are all closely associated with the progression of neurodegenerative diseases [4]. Thus, the protection of the brain from damage caused by oxidative stress can maybe alleviate neurodegenerative diseases. In addition, human neuroblastoma SH-SY5Y cells have been proven to respond to excessive ROS in a similar way to human neurons and have been often used in the studies of neurodegenerative diseases [2].

Intracellular redox homeostasis is maintained by antioxidant defense systems, such as antioxidant enzymes and signaling pathway molecules. Many efforts have demonstrated that once oxidative stress is generated, the production of antioxidant enzymes, including superoxide dismutase (SOD), catalase (CAT), and glutathione peroxidase (GPX), would be increased to restore redox homeostasis by scavenging surplus ROS [5]. In addition, the nuclear factor erythroid 2-related factor 2 (Nrf2) signaling pathway is also activated rapidly in response to cellular oxidative damage. Next, Nrf2 translocates to the nucleus and rapidly activates downstream genes, involving *heme oxygenase-1*, *glutamate-cysteine ligase catalytic subunit, and quinone oxidoreductase 1* [5,6]. Moreover, recent studies have indicated that mitogen-activated protein kinases (MAPKs) are closely related to Nrf2 nuclear translocation during the elimination of oxidative stress [7]. Although there are various antioxidant systems in cells, they are still limited to eliminating oxidative stress. Constant oxidative stress could cause cells to become dysfunctional progressively and finally die by apoptosis [8]. Therefore, more and more bioactive components, such as phenolics, carotenoids, antioxidant vitamins, and polysaccharides, were investigated to explore their potential neuroprotective properties [9,10,11,12].

Bioactive peptides are usually obtained from the hydrolysates of different protein resources and have been proven to have a variety of distinct physiological activities, for example, antioxidant, anti-cancer, antihypertensive, and other bioactivities [13]. Currently, there is a growing body of research focused on the neuroprotection of bioactive peptides. Among many kinds of compounds, peptides are suggested as good candidates for neuroprotective agents [14]. It has been reported that soybean protein hydrolysates could reverse memory and learning impairments in mice induced by D-galactose, and two antioxidant peptides (Trp-Pro-Lys and Ala-Tyr-Leu-His) from soybean protein hydrolysates could protect PC12 cells from H_2_O_2_-stimulated damage [15]. In addition, a recent study exhibited that Ser-Ser-Asp-Ala-Phe-Phe-Pro-Phe-Arg, obtained from Antarctic krill hydrolysates, was efficient in preventing scopolamine-induced oxidative stress in PC12 cells [16]. Consistently, our previous study showed that Ser-Gly-Gly-Tyr (SGGY), an antioxidant peptide obtained from walnut protein hydrolysates, played a vital role in protecting SH-SY5Y cells from cytotoxicity triggered by H_2_O_2_ [17]. However, the underlying mechanisms for the protective effects of SGGY on the H_2_O_2_-stimulated oxidative cytotoxicity of SH-SY5Y cells remain unknown. Therefore, the aim of this research was to further investigate the protective effects of SGGY on H_2_O_2_-triggered oxidative stress in SH-SY5Y cells and the underlying mechanisms accordingly.

## 2. Materials and Methods

### 2.1. Materials

SGGY (purity of >99%) was synthesized by China Peptides Co., Ltd. (Shanghai, China), which was identified from walnut protein in our previous study. Both RPMI 1640 medium and Fetal bovine serum (FBS) were supplied by Hyclone (GE Healthcare Life Sciences, Beijing, China). 3-(4,5-dimethylthiazol-2-yl)-2,5-diphenyltetrazolium bromide (MTT) was gained from MP Biomedicals Co., Ltd. (Shanghai, China). Trypsin and penicillin-streptomycin were provided by the Beyotime Institute of Biotechnology (Shanghai, China). Dimethylsulfoxide (DMSO) was gained from Sigma-Aldrich (St. Louis, MO, USA). Primary antibodies against β-actin, Bcl-2, Bax, Nrf2, JNK, ERK1/2, p38, p-JNK, p-ERK1/2 and p-p38, Histone H3 were provided by Abcam (Cambridge, UK). Horseradish peroxidase (HRP)-conjugated goat anti-rabbit IgG secondary antibody was gained from Biosynthesis Biotechnology Co., Ltd. (Beijing, China).

### 2.2. Cell Culture

SH-SY5Y human neuroblastoma cell line was supplied by the Collection of Cell Cultures of the Fourth Military Medical University (Xi’an, China). In a humidified incubator with 95% air and 5% CO_2_, the SH-SY5Y cells were grown in a complete medium comprising RPMI 1640 medium, 10% (*v/v*) FBS, and 1% (*v/v*) penicillin-streptomycin solution at 37 °C. The culture medium was changed once every two days, and the cells were collected at approximately 80% confluence for all the experiments.

### 2.3. Cell Viability Assay

The cell viability was determined by MTT assay as described previously [17]. Cells were seeded at 2 × 10^4^ cells/well into 96-well culture plates. After being incubated for 12 h, the cells were treated with SGGY (0.1, 0.5, 1, or 2 mg/mL) for 4, 8, or 12 h and subsequently stimulated by H_2_O_2_ (300, 600, 900, 1200, or 1500 μM) for 2 h. The cells of the control group were cultured in the same serum-free medium but without SGGY and H_2_O_2_ supplementation. After removing the medium with H_2_O_2_, the cells were rinsed twice with phosphate buffer solution (PBS). The MTT solution was added (final concentration 0.5 mg/mL) and the cells continued to be incubated at 37 °C for 4 h. Next, the supernatant in each well was removed and the formazan crystals formed by live cells were dissolved in each well by adding 150 μL of DMSO and shaking gently for 20 min. Absorbance of each well was determined at 490 nm by a multifunctional microplate reader (PerkinElmer Co., Boston, MA, USA). The cell viability was displayed as the percentage of the control group.

### 2.4. Lactate Dehydrogenase (LDH) Leakage Assay

LDH can be released into the medium when cell membranes are damaged. Therefore, the LDH leakage assay is usually used to evaluate the extent of cellular injury. Cells were seeded at 2 × 10^4^ cells/well into a 96-well plate and cultured for 12 h. After pretreating with SGGY (0.1, 0.5, or 1 mg/mL) for 12 h, the cells were rinsed with PBS and incubated continuously with 900 μM H_2_O_2_ for 2 h at 37 °C. The cells in the control group were incubated with the same serum-free medium but without SGGY and H_2_O_2_. Finally, the cell culture supernatant in each well was collected separately. According to the manufacturer’s instructions, the LDH activities of the supernatants were determined using a LDH assay kit (Jiancheng Institute of Biotechnology, Nanjing, China). The absorbances of samples were measured at 490 nm by a multifunctional microplate reader (PerkinElmer Co., Boston, MA, USA).

### 2.5. Reactive Oxygen Species (ROS) Assay

According to the procedure described previously [18] with some modifications, the intracellular levels of ROS in SH-SY5Y cells were determined using the fluorescent probe, 2,7-dichlorodihydrofluorescein diacetate (DCFH-DA), obtained from the Beyotime Institute of Biotechnology (Shanghai, China). Cells were seeded at 7 × 10^5^ cells per well into a 6-well plate and cultured for 12 h. After treatments as above (Section 2.4), the cells were washed with a certain amount of PBS, then the cells were incubated away from light with DCFH-DA (final concentration 10 μM) for 30 min at 37 °C. Subsequently, the cells were washed twice with PBS, lysed in cell lysis buffer containing 1% phenylmethylsulfonyl fluoride (PMSF) and 1% phosphatase inhibitor cocktail for 30 min on ice, and centrifuged at 15,000× *g* at 4 °C for 10 min. A fluorescence microscope (Olympus Optical Co., Tokyo, Japan) was used to observe the oxidative status of the cells. A fluorescence microplate reader (Molecular Devices Co., Sunnyvale, CA, USA) was used to detect the fluorescence intensities of the collected supernatants at 485 nm excitation and 535 nm emission.

### 2.6. Determination of Mitochondrial Membrane Potential (MMP)

The MMP of SH-SY5Y cells was assessed using the JC-1 probe gained from the Beyotime Institute of Biotechnology (Shanghai, China) based on the method reported previously [18]. Cells were seeded, incubated, and treated as above (Section 2.5). Next, cells were flushed twice with PBS and stained with JC-1 away from light for 30 min at 37 °C. The cells were then flushed twice with PBS. Finally, the fluorescence intensities were detected by a fluorescence microplate reader (Molecular Devices Co., Sunnyvale, CA, USA) at 538 nm FL1 (green) and 585 nm FL2 (red), and qualitatively analyzed by a fluorescence microscope (Olympus Optical Co., Tokyo, Japan).

### 2.7. Measurements of Antioxidant Enzyme Activities and MDA Level

Cells were seeded at a concentration of 2 × 10^6^ cells per well in 60 mm dishes and cultured at 37 °C for 12 h. After treatments as above (Section 2.4), the cells were collected. The cells were then flushed with PBS. Finally, according to the instructions, the activities of SOD, MDA, and CAT in SH-SY5Y cells were assessed by commercial kits from the Jiancheng Bioengineering Institute (Nanjing, China).

### 2.8. Analysis of Apoptosis

Cell apoptosis was evaluated by an Annexin V-FITC/PI apoptosis detection kit purchased from the Beyotime Institute of Biotechnology (Shanghai, China). SH-SY5Y cells in 6-well plates with different treatments were trypsinized, collected, centrifuged, and flushed twice with ice-cold PBS sequentially. The cells were resuspended in 200 μL binding buffer and then incubated with staining solution made of 5 μL Annexin V-FITC and 10 μL PI away from light for 10 min at room temperature. Finally, the stained cells were monitored with a FACSAria (BD Biosciences, Franklin Lakes, NJ, USA) immediately and analyzed by Flowjo 7.6 software.

### 2.9. Western Blot Analysis

SH-SY5Y cells were plated at 2 × 10^6^ cells per well in 60 mm dishes and cultured at 37 °C for 12 h. After treatments, the proteins in the cells were extracted for Western blot analysis. After the cells were flushed twice with PBS, they were collected and lysed in RIPA lysis buffer containing 1% PMSF and 1% phosphatase inhibitor cocktail for 30 min on ice to obtain total proteins. Then, the lysate was centrifuged at 15,000× *g* for 10 min at 4 °C. Nuclear and cytoplasmic proteins were extracted with a nuclear and cytoplasmic protein extraction kit in accordance with manufacturer’s guidelines supplied by the Beyotime Institute of Biotechnology (Shanghai, China). The protein concentration was determined by a bicinchoninic acid (BCA) protein assay kit from Hat Biotechnology Co., Ltd. (Xi’an, China). Western blot analysis was performed with some modifications according to the previous report [19]. The proteins were resolved by sodium dodecyl sulfate polyacrylamide gel electrophoresis (SDS-PAGE) and then transferred onto polyvinylidene fluoride (PVDF) membranes. The membranes were blocked with 5% skim milk in TBST (50 mM Tris-HCl; pH 7.4; 150 mM NaCl; 0.1% Tween-20) and kept at room temperature for 1 h. After rinsing once with TBST, the membranes were incubated with primary antibodies overnight at 4 °C. Subsequently, the membranes were washed 3 times with TBST followed by incubations with secondary antibodies for 2 h at room temperature and 3 times washing with TBST. Finally, immunoreactive proteins in the membranes were visualized with an ECL commercial kit by a Chemiluminescence imaging system (Sage creation Science, Beijing, China).

### 2.10. Statistical Analysis

All quantitative results from three independent determinations were expressed as means ± standard deviation (SD). The significance of differences among data was analyzed using one-way ANOVA and the least significant difference (LSD) by SPSS 18.0 (*p* < 0.05).

## 3. Results

### 3.1. SGGY Suppressed H_2_O_2_-Induced Cytotoxicity in SH-SY5Y Cells

**Figure 1:**
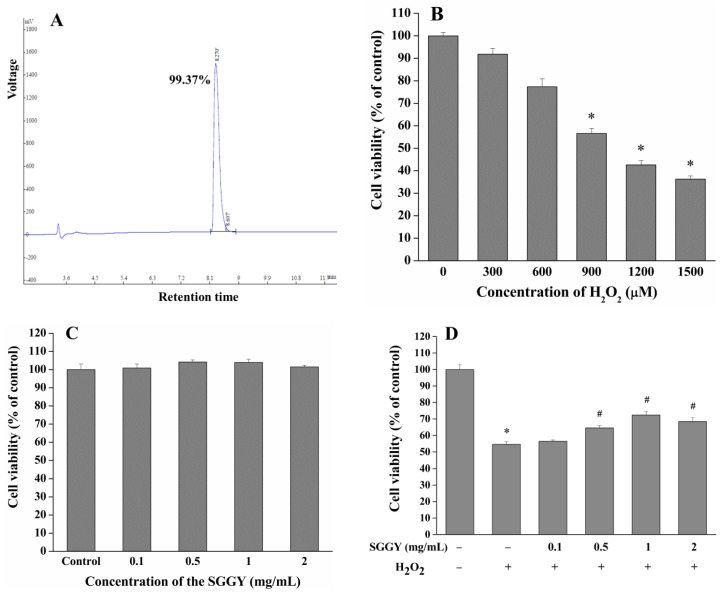

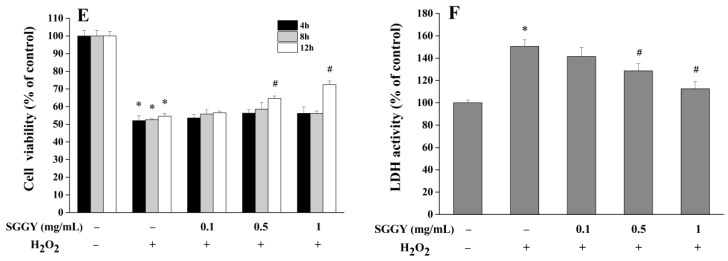
SGGY suppressed H_2_O_2_-triggered cytotoxicity in SH-SY5Y cells. (**A**) Purity of SGGY. Cells were treated for 2 h with (**B**) various concentrations of H_2_O_2_, or for 12 h with (**C**) different concentrations of SGGY, and subsequently, the cell viability was determined using the MTT assay. Cells were pre-incubated with (**D**) various concentrations of SGGY for 12 h or (**E**) indicated concentrations of SGGY for various times, and subsequently exposed to 900 μM H_2_O_2_ for 2 h followed by a determination of the cell viability. (**F**) Cells were pre-incubated with indicated concentrations of SGGY for 12 h, and subsequently exposed to 900 μM H_2_O_2_ for 2 h; the leakage of LDH was measured by a LDH detection kit. * *p* < 0.05 vs. control group, and # *p* < 0.05 vs. H_2_O_2_ group.

In our previous study, an antioxidant peptide was identified as SGGY from walnut protein hydrolysate and has been suggested to possess the potential for neuroprotection. SGGY was synthesized by a solid-phase peptide synthesizer and its purity was 99.37% (Figure 1A). To establish an oxidative injury model, SH-SY5Y cells were exposed to a range of concentrations of H_2_O_2_ (300, 600, 900, 1200, and 1500 μM) for 2 h. The results indicated that H_2_O_2_ treatment significantly reduced the viability of SH-SY5Y cells (56.6%) when its concentration increased to 900 μM (*p* < 0.05, Figure 1B). Therefore, the optimal concentration of H_2_O_2_ was chosen to be 900 μM in our subsequent experiments. As shown in Figure 1C,D, SGGY could protect SH-SY5Y cells from cytotoxicity stimulated by H_2_O_2_ in a dose-dependent manner, with no apparent cytotoxicity up to a concentration of 2 mg/mL. Cell viability of SH-SY5Y was significantly increased by SGGY pretreatment and there was no obvious difference in the protective effects between SGGY concentrations of 1 and 2 mg/mL. Therefore, SGGY concentrations of 0.1, 0.5, and 1 mg/mL were selected to investigate the protective effect of SGGY on SH-SY5Y cells in subsequent experiments. To further determine the impacts of treatment time on cell viability, SH-SY5Y cells were pre-incubated with SGGY (0.1, 0.5, and 1 mg/mL) for 4, 8, and 12 h, respectively. The cells were then exposed to 900 μM H_2_O_2_ for 2 h. The results indicated that SGGY could protect SH-SY5Y cells against H_2_O_2_-triggered cytotoxicity, showing a dose-dependent manner, only when the pre-incubation time reached 12 h (Figure 1E). Therefore, the optimal pre-incubation time was chosen to be 12 h in subsequent experiments. As shown in Figure 1F, SGGY pretreatment prevented the leakage of LDH induced by H_2_O_2_, which is obviously associated with cell membrane integrity, and this was shown to be in a dose-dependent manner.

### 3.2. SGGY Inhibited H_2_O_2_-Stimulated Apoptosis in SH-SY5Y Cells

**Figure 2:**
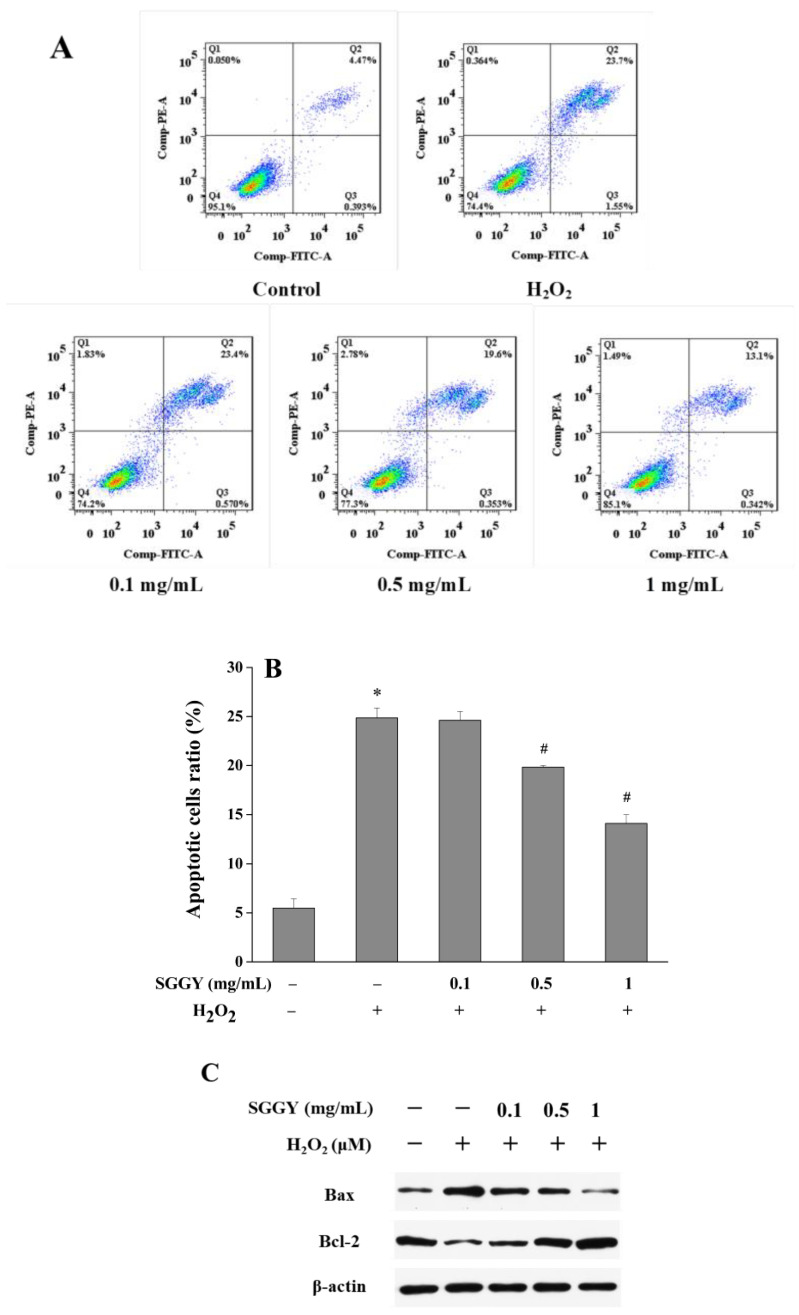
SGGY inhibited H_2_O_2_-stimulated apoptosis in SH-SY5Y cells. Cells were pre-incubated with SGGY (0.1, 0.5, and 1 mg/mL) for 12 h and subsequently exposed to 900 μM H_2_O_2_ for 2 h. (**A**) Apoptosis was analyzed using Annexin V-FITC/PI staining by flow cytometry. The graphs contain the percentage of necrotic cells (Q1), late apoptotic cells (Q2), early apoptotic cells (Q3), and normal cells (Q4) relative to the total cells. **(B**) Quantitative analysis of the apoptosis ratio of early and late apoptotic cells. (**C**) The expression of Bcl-2 and Bax were explored by Western blotting. * *p* < 0.05 vs. control group, and # *p* < 0.05 vs. H_2_O_2_ group.

As shown in Figure 2A,B, compared to the control, the ratio of total apoptosis (combining the early and late apoptotic cells) increased dramatically after H_2_O_2_ treatment (*p* < 0.05). However, SGGY pre-treatment could protect SH-SY5Y cells against cell apoptosis triggered by H_2_O_2_. Pre-incubation with SGGY (0.1, 0.5, and 1 mg/mL) reduced the percentage of total apoptosis to 24.61%, 19.84%, and 14.10%, respectively, showing a dose-dependent manner. Specifically, 0.5 and 1 mg/mL SGGY supplementations exhibited a significant protective effect against SH-SY5Y cell apoptosis stimulated by H_2_O_2_ (*p* < 0.05). In addition, H_2_O_2_ treatment upregulated the expression of Bax and downregulated that of Bcl-2 in SH-SY5Y cells, thus the ratio of Bax/Bcl-2 was raised compared to the control. However, pre-incubation with SGGY (0.1, 0.5, and 1 mg/mL) dramatically reduced the ratio of Bax/Bcl-2, presenting a dose-dependent manner (Figure 2C).

### 3.3. SGGY Ameliorated H_2_O_2_-Induced Redox Imbalances in SH-SY5Y Cells

**Figure 3:**
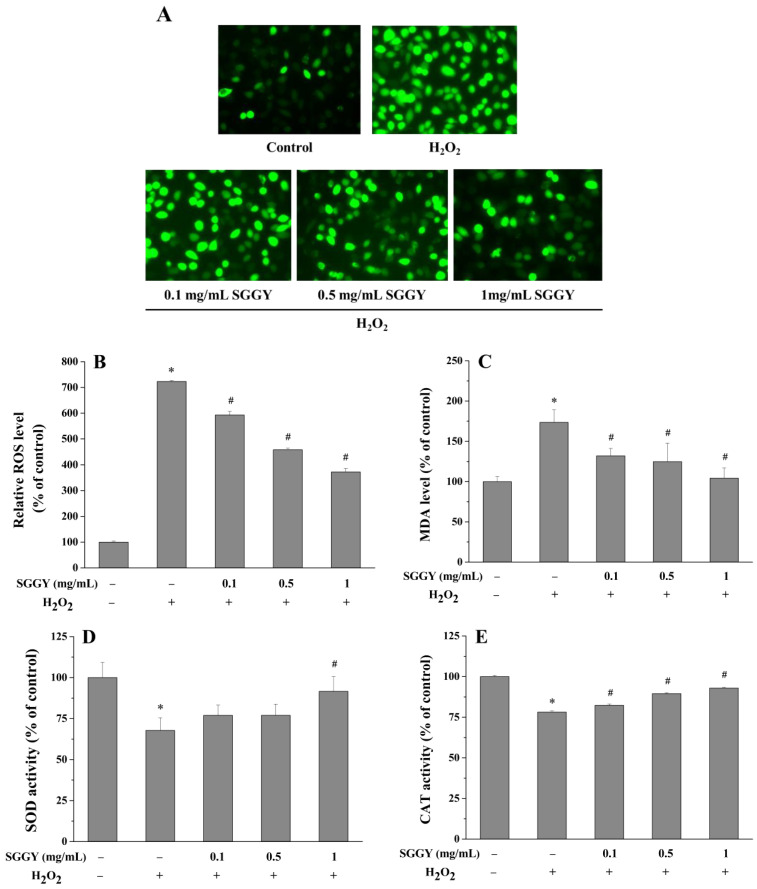
SGGY suppressed H_2_O_2_-triggered redox imbalances in SH-SY5Y cells. Cells were pre-incubated with indicated concentrations of SGGY (0.1, 0.5, and 1 mg/mL) for 12 h and subsequently exposed to 900 μM H_2_O_2_ for 2 h. After staining with DCFH-DA, the oxidative status of the cells was visualized by a fluorescence microscope. The green colors represented the DCFH-DA fluorescence intensity in SH-SY5Y cells. The brighter the green colors, the stronger the fluorescence intensity. (**A**); the fluorescence intensity was detected by a fluorescence microplate reader. (**B**) Relative level of intracellular ROS. MDA (**C**), SOD (**D**) and CAT (**E**) levels in SH-SY5Y cells were detected. * *p* < 0.05 vs. control group, and # *p* < 0.05 vs. H_2_O_2_ group.

Compared to the control group, the level of intracellular ROS in SH-SY5Y cells was significantly upregulated after exposure to H_2_O_2_ (*p* < 0.05; Figure 3A,B). However, pre-incubation of cells with SGGY (0.1, 0.5, and 1 mg/mL) effectively reduced intracellular ROS, presenting a dose-dependent manner (*p* < 0.05). The contents of MDA, SOD, and CAT closely related to the cellular redox status were also determined. After being exposed to H_2_O_2_, the MDA level was increased to approximately 1.75-fold of the control (*p* < 0.05; Figure 3C), while the activities of SOD and CAT were dramatically reduced compared to the control (*p* < 0.05; Figure 3D,E). However, SGGY pre-incubation could significantly decrease the MDA content and increase the CAT activity of SH-SY5Y cells in a dose-dependent manner, except for SOD activity, which only showed a significant increase by 1 mg/mL SGGY pre-incubation (*p* < 0.05; Figure 3C–E). The results indicated that SGGY possessed strong antioxidant activity to maintain redox homeostasis in SH-SY5Y cells.

### 3.4. SGGY Ameliorated H_2_O_2_-Induced Mitochondrial Dysfunction in SH-SY5Y Cells

**Figure 4:**
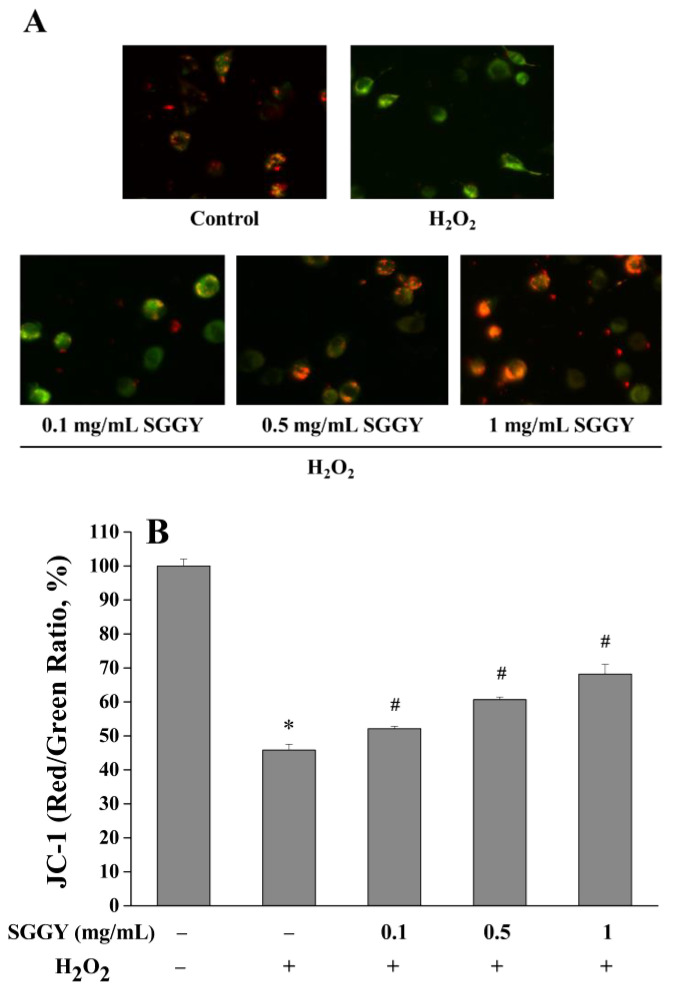
SGGY ameliorated H_2_O_2_-induced mitochondrial dysfunction in SH-SY5Y cells. Cells were pre-incubated with different concentrations of SGGY (0.1, 0.5, and 1 mg/mL) for 12 h and subsequently exposed to 900 μM H_2_O_2_ for 2 h. After being incubated with JC-1, the changes in MMP were visualized by a fluorescence microscope. The red colors represented normal mitochondrial membrane potential in SH-SY5Y cells, and the green colors represented that mitochondrial membrane potential was decreased. (**A**); the fluorescence intensities of JC-1 were measured by a fluorescence microplate reader (**B**). * *p* < 0.05 vs. control group, and # *p* < 0.05 vs. H_2_O_2_ group.

To evaluate the protective effect of SGGY on mitochondrial function in SH-SY5Y cells, MMP, an early indicator of alteration in mitochondrial function, was determined by JC-1 staining. As shown in Figure 4A, fluorescence microscope observations showed that pretreatment of SGGY with SH-SY5Y cells reduced the loss of MMP stimulated with H_2_O_2_. Similarly, compared to the control group, fluorescence quantitative results also demonstrated that H_2_O_2_ treatment resulted in the decline of MMP significantly (*p* < 0.05), whereas pre-incubation of cells with SGGY effectively prevented the decline of MMP, showing a dose-dependent manner (*p* < 0.05; Figure 4B).

### 3.5. SGGY Activated the Nrf2 Signaling Pathway in SH-SY5Y Cells

**Figure 5:**
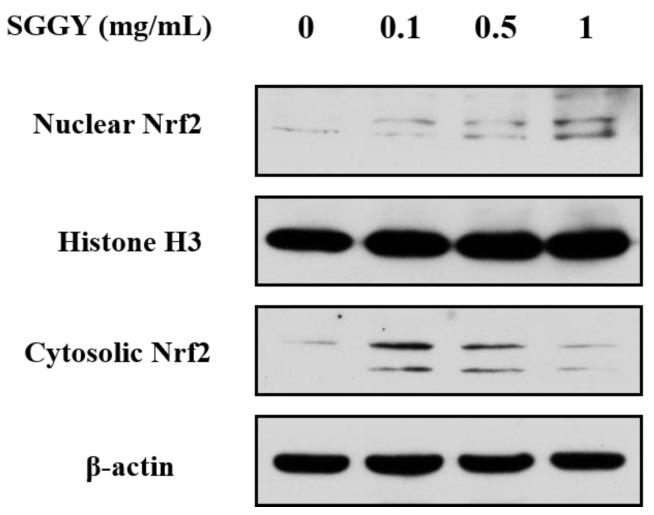
SGGY activated the Nrf2 of SH-SY5Y cells. Cells were pre-incubated for 12 h with indicated concentrations of SGGY (0.1, 0.5, and 1 mg/mL). The nuclear translocation of Nrf2 was detected by Western blotting.

In order to understand the role of SGGY in the protective mechanisms against the cytotoxicity of SH-SY5Y cells triggered by H_2_O_2_, the influence of SGGY on the Nrf2 signaling pathway was detected by Western blotting. The results suggested that the nuclear translations of Nrf2 in SH-SY5Y cells were significantly promoted and that cytosolic levels of Nrf2 were decreased accordingly after SGGY treatment, presenting a dose-dependent manner (Figure 5).

### 3.6. SGGY Inhibited H_2_O_2_-Stimulated Activation of the MAPKs Signaling Pathway in SH-SY5Y Cells

**Figure 6:**
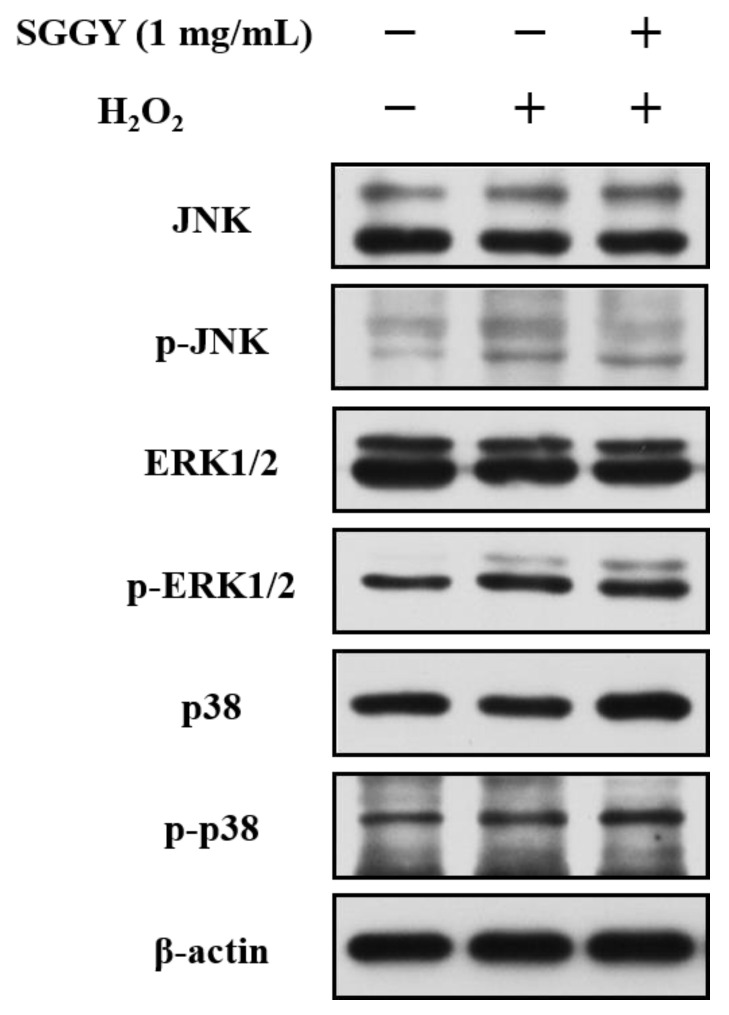
SGGY inhibited H_2_O_2_-triggered activation of the MAPKs signaling pathway in SH-SY5Y cells. Cells were pre-incubated with 1 mg/mL SGGY for 12 h and subsequently exposed to 900 μM H_2_O_2_ for 2 h. The expression of JNK, p-JNK, ERK1/2, p-ERK1/2, p38 and p-p38 were measured by Western blotting.

To further investigate the effect of SGGY on MAPK signal transduction in SH-SY5Y cells triggered by H_2_O_2_, we detected the phosphorylation levels of JNK, ERK1/2, and p38. As shown in Figure 6, JNK, ERK1/2, and p38 MAPKs were significantly activated by H_2_O_2_. The ratios of p-JNK/JNK, p-ERK/ERK, and p-p38/p38 were also raised. An amount of 1 mg/mL SGGY could inhibit the H_2_O_2_-stimulated JNK and p38 activation. However, there was no remarkable change in the p-ERK/ERK ratios between the H_2_O_2_ and H_2_O_2_+SGGY groups.

## 4. Discussion

Recently, peptides derived from food materials have attracted more and more attention for their better biological activities than proteins. Walnuts have been well documented as a natural source of high-quality proteins. Bioactive peptides derived from walnut protein have been suggested to possess various biological functions, such as ACE-inhibitory activity, antioxidant activity, and anti-cancer activity, which were attributed to the amino acid compositions and sequences [17,20]. SGGY, a novel bioactive peptide identified from walnut protein, has been proven to possess strong antioxidant activity in our previous study, suggesting that it may have the potential to relieve oxidative damage to cells [17]. Therefore, the protective effects of SGGY against oxidative stress stimulated by H_2_O_2_ in SH-SY5Y cells and the potential mechanisms were explored in this work.

Mitochondria are the major organelles for regulating energy transduction and redox homeostasis in cells, and MMP changes are considered to be indicative of mitochondrial dysfunction [21]. Many studies have demonstrated that H_2_O_2_ could trigger mitochondrial dysfunction and alter the MMP values in cell models, resulting in deleterious effects caused by the production of intracellular ROS [22]. Furthermore, excessive ROS could attack the mitochondria in turn, giving rise to a vicious cycle of increased mitochondrial ROS production, which finally results in serious neuronal dysfunction and even neurodegeneration [23,24]. In addition, the high level of ROS has been reported to be related to many pathological conditions, such as cancer, diabetes, and other chronic diseases [25]. In this study, the results indicated that H_2_O_2_ obviously reduced the cell viability and, on the other hand, raised the leakage of LDH in the culture medium, suggesting that the integrity of the cell membrane was damaged by H_2_O_2_ and finally resulted in cell death (Figure 1D,F). In addition, intracellular ROS levels were significantly increased and MMP was remarkably lost after cells were exposed to H_2_O_2_ (Figure 3A,B and Figure 4). However, SGGY treatment notably reversed the intracellular ROS levels, prevented the decline of MMP, ameliorated the leakage of LDH, and increased cell viability. Wang et al. [26] also stated that antioxidant peptides (WSREEQEREE and ADIYTEEAGR) derived from walnut could inhibit ROS production, leakage of LDH, and MMP collapse caused by glutamate in PC12 cells. In addition, a previous study has demonstrated that an antioxidant peptide (WLP) derived from the sea squirt effectively suppressed cytotoxicity in 6-Hydroxydopamine-induced PC12 cells by increasing cell viability and decreasing intracellular ROS levels in a dose-dependent manner [27]. However, not all antioxidant peptides exhibited protective effects, which might be attributed to their structural characteristics, such as amino acid sequence, composition, and hydrophobicity [17,28]. More importantly, SGGY was still nontoxic to SH-SY5Y cells when the concentration was up to 2 mg/mL, which was consistent with the view that food-derived peptides are generally recognized as safe [29].

Apoptosis, a form of programmed cell death, is also considered to be associated with the accumulation of ROS [30]. The Bcl-2 family proteins are important signaling molecules that play a critical role in regulating cell apoptosis, among which Bcl-2 and Bax are major members [31]. Bcl-2, an anti-apoptotic protein, inhibits apoptosis by diminishing ROS [32]. In contrast, the pro-apoptotic protein Bax could promote apoptosis by translocating into the mitochondrial membrane and then modifying the selective permeability of ions across the mitochondrial membrane [33]. Therefore, the ratio of Bax/Bcl-2 is usually regarded as a predictive marker to illuminate the ability of cells to fight against apoptosis [8]. In this study, we investigated whether Bax and Bcl-2 were involved in the apoptosis of H_2_O_2_-stimulated SH-SY5Y cells pretreated with or without SGGY. The result showed that exposure to H_2_O_2_ significantly upregulated the ratio of Bax/Bcl-2, while SGGY pre-incubation decreased the ratio of Bax/Bcl-2 and it presented a dose-dependent manner (Figure 2C), which agrees with the findings of cell viability and apoptosis. Similar results were reported by Wang et al. [26], who found that walnut-derived peptides exerted neuroprotective effects on cell apoptosis by up-regulating the expression of Bax and down-regulating the expression of Bcl-2 in PC12 cells.

Antioxidant enzymes could effectively inhibit apoptosis by eliminating excessive amounts of ROS and maintaining cellular physiological functions [30]. In the antioxidant enzyme system, SOD is responsible for catalyzing the superoxide radical with high cytotoxicity and strong oxidation into hydrogen peroxide or ordinary molecular oxygen, while CAT catalyzes hydrogen peroxide into harmless water and oxygen directly [34]. In this study, we found that exposure to H_2_O_2_ dramatically weakened the activities of SOD and CAT in SH-SY5Y cells, while increasing them after SGGY pretreatment (Figure 3D,E). Consistent with these results, the content of MDA, regarded as a marker of oxidative stress, was raised after exposure to H_2_O_2_, and SGGY significantly decreased the MDA content in SH-SY5Y cells (Figure 3C). Our results indicated that the underlying protective mechanisms of SGGY against oxidative stress were closely associated with the antioxidant defense system of SH-SY5Y cells. The protective effect of SGGY on the antioxidant defense system was consistent with that of walnut protein hydrolysates, which have been reported to be capable of attenuating the scopolamine-induced abnormal antioxidant defense system in mouse brains by increasing the activities of SOD, GSH-px, and CAT, and decreasing the content of MDA [35]. Additionally, the same result has been reported by Mates et al., that the bioactive compounds composed of phenolic compounds, lipids, carbohydrates, amino acids, etc., in walnut septum extract could replenish the antioxidant defense system by decreasing the levels of ROS, MDA, and other oxidative stress biomarkers [36]. Among the bioactive compounds, a low molecular weight polysaccharide (DJP-2) has been proven to have a protective effect against H_2_O_2_-induced oxidative damage in cells [37].

In addition to the antioxidant enzymes, Nrf2 is also crucial to improving the antioxidant capacity of cells [38]. In this study, as shown in Figure 5, SGGY significantly promoted the nuclear translations of Nrf2 in SH-SY5Y cells, indicating that the protective effects of SGGY on SH-SY5Y cells were related to the activation of Nrf2. This result was consistent with the previous report that peptides could activate the Nrf2-Keap1 signaling pathway [39]. A recent study also found that a strong interaction existed between walnut-derived peptides and Keap1 by the method of molecular docking [26]. The MAPKs signaling pathway has been recognized as the upstream signal of Nrf2 activation [40]. In this study, we also investigated the activation of MAPKs (p38, ERK, and JNK) by Western blotting. The findings suggested that SGGY attenuated the H_2_O_2_-stimulated phosphorylation of JNK and P38 but had no impact on the activation of ERK (Figure 6). The results are consistent with a previous report that emodin inhibited the activation of JNK and P38 but had no influence on the LPS-Stimulated phosphorylation of ERK [40]. This phenomenon is probably because the JNK and p38 pathways associated with cell apoptosis were persistently activated by H_2_O_2_, however, the phosphorylation of ERK was increased in the early stage and then decreased [41]. These findings demonstrated that Nrf2, JNK, and P38 may participate in the protective effects of SGGY against the H_2_O_2_-triggered oxidative stress of SH-SY5Y cells.

**Figure 7:**
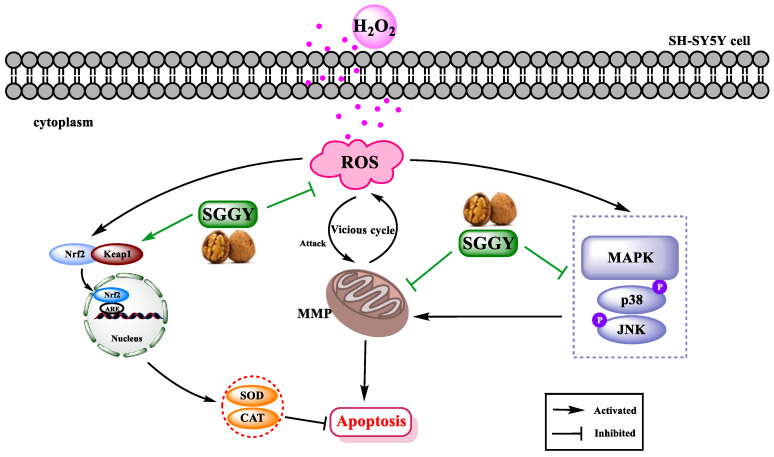
Molecular mechanism of SGGY-suppressed H_2_O_2_-induced SH-SY5Y cells’ cytotoxicity. H_2_O_2_ increases the level of intracellular ROS, and then the oxidative stress induced by H_2_O_2_ triggers the JNK, p38, and Nrf2 signaling pathways. While SGGY pre-treatment attenuates the generation of intracellular ROS and dysfunction of mitochondria, it suppresses the phosphorylation of JNK and p38 and promotes the nuclear translations of Nrf2 to improve the antioxidant capacity of cells, thereby enhancing the activity of cells. The arrows in green highlight the primary roles of SGGY in protecting SH-SY5Y cells from H_2_O_2_-stimulated oxidative stress.

In conclusion, SGGY is a natural antioxidant peptide identified from walnut protein and has the capacity of ameliorating oxidative stress as well as a series of neuron dysfunctions provoked by H_2_O_2_ in SH-SY5Y cells. This study suggested that the underlying neuroprotective mechanism of SGGY may be to eliminate free radicals by improving the antioxidant defense system, which may be associated with the decline in the phosphorylation of JNK and p38 and the nuclear translations of Nrf2 (Figure 7). Furthermore, our results suggested that SGGY might be applied as a candidate for dietary supplements to maintain brain health. More efforts will be necessary to further uncover the functions and protective mechanisms of SGGY on brain health in vitro and in vivo.

## Data Availability

The data used to support the findings of this study can be available by the corresponding author upon request.
